# The Combined Effect of Safety Specific Transformational Leadership and Safety Consciousness on Psychological Well-Being of Healthcare Workers

**DOI:** 10.3389/fpsyg.2021.688463

**Published:** 2021-06-21

**Authors:** Muhammad Irshad, Mehwish Majeed, Sana Aroos Khattak

**Affiliations:** ^1^Faculty of Management Sciences, National University of Modern Languages Islamabad Campus, Islamabad, Pakistan; ^2^Faculty of Management Sciences, International Islamic University, Islamabad, Pakistan; ^3^Faculty of Management Sciences, Bahria University Islamabad, Islamabad, Pakistan

**Keywords:** safety specific transformational leadership, psychological well-being, safety consciousness, healthcare worker, occupational hazard, COVID-19 perceived risk

## Abstract

Occupational health researchers have begun to realize that the psychological well-being of healthcare workers who are providing treatment against COVID-19 is deteriorating. However, there is minimal research conducted on it, particularly in the context of leadership. The current study aims to fill this important gap by identifying critical factors that can enhance the psychological well-being of healthcare workers. We proposed that safety specific transformational leadership enhances psychological well-being among healthcare workers, and COVID-19 perceived risk mediates this relationship. Furthermore, the safety conscientiousness of healthcare workers was proposed to be a boundary condition that enhances the negative relationship between safety-specific transformational leadership and COVID-19 perceived risk. Data were collected from healthcare workers (*N* = 232) treating COVID-19 patients in the hospitals of Pakistan through well-established adopted questionnaires. The discriminant and convergent validity of the data was tested through confirmatory factor analysis by using AMOS statistical package. The mediation and moderation hypotheses were tested by using PROCESS Macro by Hayes. The results showed that safety specific transformational leadership enhances psychological well-being among healthcare workers, and COVID-19 perceived risk mediates this relationship. Moderation results also confirmed that safety conscientiousness moderates the relationship between safety specific transformational leadership and COVID-19 perceived risk. This study offers implications for both researchers and practitioners.

## Introduction

The world is facing one of the worst pandemics in the history of mankind (Balkhair, 2020). The COVID-19 infection has affected people in general and employees, particularly as they have an additional safety hazard whirling around them (Yu et al., 2021). Despite the enforcement of lockdown and curfew in most parts of the world, healthcare workers are bound to be physically present at the workplace due to the nature of their job, which has raised a serious concern for their physical and psychological health (Wilson et al., 2020). Multiple studies conducted on healthcare workers during the last year have indicated an increase in anxiety, psychological distress, depression, and various other mental health issues (Raudenská et al., 2020; Shechter et al., 2020; Lenzo et al., 2021). Many healthcare workers have lost their lives to COVID-19 while treating the infected patients that have generated a wave of fear among the healthcare workers (Apisarnthanarak et al., 2020; Kumar et al., 2020; Lapolla et al., 2021). Healthcare workers are concerned for their lives and are looking toward their leaders, hoping to develop and ensure safety measures in the hospitals.

In this critical time, the role of leadership cannot be ignored (Billings et al., 2020; Sant'Ana et al., 2020; Zhao et al., 2020a). While studies have shown that transformational leadership, inclusive leadership and servant leadership seems to be a suitable leadership style for managing employees working in education sector during this pandemic, there is no evidence on the effectiveness of these leadership styles in hospitals settings and occupational safety offered by these leadership styles (Azizaha et al., 2020; Fournier et al., 2020; Zhao et al., 2020b; Antonopoulou et al., 2021; Piorun et al., 2021). There is an urgent need to implement a leadership style that has a prime focus on the occupational safety of employees so that healthcare workers may feel safe in the hospitals while serving the COVID-19 patients (Labrague and De los Santos, 2020; Rosa et al., 2020; Zhao et al., 2020b).

According to the limited literature available on safety leadership, safety specific transformational leaders are suitable for occupations with higher occupational hazards (de Koster et al., 2011; Smith et al., 2020). What makes them different from conventional transformational leaders is their extra emphases on employee safety (Barling et al., 2002; Willis et al., 2017). They encourage employees to look for more effective ways of ensuring safety (intellectual stimulation), inspire them to achieve safety standards with were considered unattainable in the past (inspirational motivation), promote occupational safety as a core value (idealized influence), and take a keen interest in the physical and mental well-being of every single employee (individual consideration; Barling et al., 2002; Smith et al., 2020).

We believe that healthcare workers working under safety specific transformational leadership are less likely to develop COVID-19 perceived risk due to all the additional occupationally safety measures taken by their leader. This is particularly true for those healthcare workers who have safety consciousness mainly because they are themselves concerned and mindful about their safety (de Koster et al., 2011). Safety consciousness is different from consciousness personality trait as it involves awareness about safety rather than general awareness and consciousness. Safety consciousness research is only limited to those organizations which carries a high risk of occupational hazards (Chun et al., 2018; Khan et al., 2018) while ignoring its utility in the context of a pandemic. Since hospitals dealing with COVID-19 patients also pose occupational hazards to the healthcare workers, it is important to investigate its moderating role in hospitals providing treatment for COVID-19 infection. Employees with safety consciousness are more careful about their and others' safety while carrying out their routine tasks (Lee, 2017; Khan et al., 2018), which is crucial for containing the COVID-19 virus. Hence, we believe that a safety transformational leader together with safety-conscious employees helps in mitigating COVID-19 perceived risk up to a great extent.

The primary rationale for choosing employee perception instead of emotions as an underlying mechanism is the scarcity of research on its antecedents and outcomes and a repeated call for studying it in the context of COVID-19 (Lam et al., 2020; Shin and Kang, 2020). COVID-19 perceived risk has recently emerged as an essential factor that is deemed responsible for a wide range of adverse employee outcomes (Lam et al., 2020; Yildirim and Güler, 2021). Keeping in view its significance, there has been a repeated call for identifying the antecedents and consequences of COVID-19 perceived risk (Bae and Chang, 2020; Lam et al., 2020; Shin and Kang, 2020). The existing literature available on COVID-19 perceived risk has identified its detrimental outcomes for employees, particularly healthcare workers (Gorini et al., 2020; Yildirim et al., 2020). According to some studies, it is the root cause behind an increase in mental health issues among healthcare workers and needs immediate attention from occupational health researchers (Alsubaie et al., 2019; Chu et al., 2021; Yildirim et al., 2021).

The literature on COVID-perceived risk has only identified its adverse outcomes (for reference see (Alsubaie et al., 2019; Gorini et al., 2020; Yildirim et al., 2020, 2021; Chu et al., 2021), there is a scarcity of research on the factors that can minimize COVID-19 perceived risk among employees. It is crucial to identify factors that can decrease COVID-19 perceived risk among healthcare workers. We propose that safety specific transformational leadership can significantly reduce COVID-19 perceived risk among healthcare workers due to its focus on the occupational safety of healthcare workers.

Research indicates that the stress and anxiety level of employees increases with an increase in COVID-19 perceived risk (Lam et al., 2020; Yan et al., 2020; Yildirim and Güler, 2021). The extant research on psychological and mental well-being suggest that employees satisfied with the preventive measures taken by their organization are less vulnerable to mental health issues during COVID-19 (Ahmed et al., 2020; Bashirian et al., 2020; Wee et al., 2020; Zhao et al., 2020b). Despite the plethora of studies conducted on the well-being of employees, there is still a need to identify the role of leadership in enhancing employee well-being during COVID-19 (Dirani et al., 2020; Zhao et al., 2020b; Haque, 2021). We believe that safety specific transformational leadership in combination with employee safety consciousness decreases COVID-19 perceived risk among employees, ultimately improving their psychological well-being. The major rationale for focusing on the employee psychological outcomes of safety specific transformational leadership is the repeated call for studying the antecedents of psychological outcomes among employees during COVID-19 (Wee et al., 2020; Zhao et al., 2020b). A vast amount of studies has highlighted an increase in psychological health issues among employees during COVID-19 (Lam et al., 2020; Yan et al., 2020). It is crucial to investigate the factors that can enhance the psychological well-being of employees during COVID-19 (Gavin et al., 2020). Some researchers have particularly identified the need to investigate the factors that can enhance psychological well-being among healthcare workers (Chew et al., 2020; Gavin et al., 2020; Greenberg et al., 2020; Tan et al., 2020).

The proposed model gets its support from the high reliability organizational theory (Roberts, 1990; La Porte, 1996), which states that organizations can minimize occupational hazards by taking sufficient safety measures. This theory states that high reliability organizations minimize occupational risks by engaging in activities that promote employee safety, which is beneficial for employees (Ford, 2018). We believe that transformational leaders and safety-conscious employees decrease COVID-19 perceived risk among employees, enhancing their psychological well-being.

**Figure 1:**
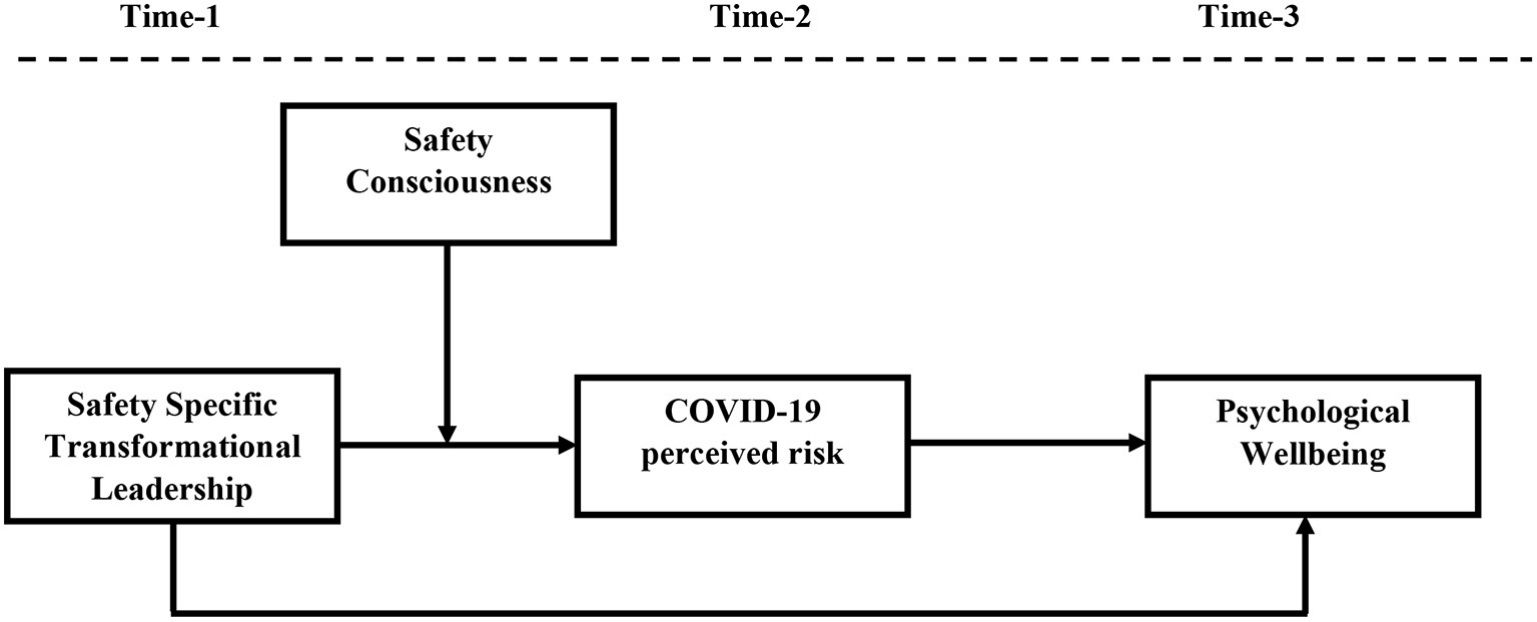
Research model. Shows proposed model where safety specific transformational leadership enhances psychological well-being of employees by reducing COVID-19 perceived risk at different levels of safety consciousness.

To summarize, the current study investigates the impact of safety specific transformational leadership on the psychological well-being of healthcare workers by taking into account the mediating role of COVID-19 perceived risk. The current study also aims to test safety consciousness as a boundary condition that strengthen the negative relationship between safety specific transformational leadership and COVID-19 perceived risk. Figure 1 shows the proposed theoretical framework.

## Theory and Hypothesis Development

### Supporting Theory

The current study is developed under the lens of high reliability organizational theory (Roberts, 1990; La Porte, 1996). According to this theory, irrespective of the complexity of organizational tasks, highly reliable organizations minimize occupational hazards and maximize employee safety by strictly following safety protocols, helping them create a safe workplace for employees (Roberts and Bea, 2001; de Koster et al., 2011). This theory defines highly reliable organizations are those, which continuously strive to increase safety by focusing on all aspects of the tasks, going out of the way to implement safety measures, practice resilience by providing safety training to employees and getting regular feedback from employees on how to improve safety procedures (Veazie et al., 2019). This theory mainly focuses on complex work units in which hazards are inevitable (Wolf, 2001). The high reliability organizational theory states that employees working under a high reliability organization are less likely to experience physical and mental health issues (Agwu et al., 2019).

We believe that safety specific transformational leadership, due to its enhanced focus on safety combined with employee safety consciousness, make hospitals high reliability organizations by minimizing COVID-19 perceived risk and improving the psychological well-being of healthcare workers. Since hospitals are fairly complex work units that involve technical work, the high reliability organizational theory can apply to them.

This theory has frequently been used in studies conducted on occupational hazards and employee safety (Roberts and Bea, 2001; de Koster et al., 2011). A careful review of existing literature on high reliability organizational theory suggests that it has frequently been used in the healthcare, nuclear industries, construction industry, aerospace, and oil and gas industry mainly because these industries pose a high occupational risk and require additional measures to ensure the safety of employees (Enya et al., 2018). Researchers believe that those organizations that emphasize safety and go to extreme lengths to avoid risks are deemed high reliability organizations (Thomassen et al., 2011). Since safety transformational leadership and safety consciousness focus on safety and risk avoidance and help minimize perceived risk, we suggest that high reliability organizational theory supports our proposed model.

### Safety Specific Transformational Leadership and Employee Psychological Well-Being

Research on safety specific transformational leadership is gaining attention mainly because of its positive outcomes for employees exposed to occupational hazards (Barling et al., 2002; de Koster et al., 2011). Safety specific transformational leadership focuses on employee safety through inspirational motivation, idealized influence, intellectual stimulation, and individual consideration (Smith et al., 2020). Inspirational motivation enables safety specific transformational leaders to motivate employees and encourage them to achieve those safety standards deemed unattainable (Mullen and Kelloway, 2009). The idealized influence allows leaders to become role model in promoting safety by developing safety as a core value (Conchie and Donald, 2009). Intellectual stimulation helps safety specific transformational leaders enhance employee safety by encouraging employees to find new and better ways to ensure safety (Smith et al., 2016). Lastly, individual consideration covers the supervisor-follower relationship by stating that safety specific leaders show great concern for their employees' physical safety and overall well-being (de Koster et al., 2011). Altogether, these four core components of safety specific transformational leadership give employees the message that their leaders care for them, is concerned about their well-being and are willing to extra miles to make them feel safe at work (Smith et al., 2020). This may enhance psychological well-being among employees by making them feel safe from occupational hazards (Johnson, 2019).

The extant research also supports the positive association with positive forms of leadership and psychological well-being of employees (Arnold, 2017; Park et al., 2017; Inceoglu et al., 2018). Safety specific transformational leaders maintain good relation with employees by giving individual consideration; they also give autonomy to the employee by asking them to find ways for improving safety standards (Johnson, 2019). Also, they provide a safe workplace to employees where they can grow and prosper (Smith et al., 2020). Taken together, safety specific transformational leadership promotes autonomy among employees, enables their personal growth, and develops good relations with them, all of which are essential components of psychological well-being (Barling et al., 2002; Willis et al., 2017). Hence, we propose that safety specific leadership leads to an increase in the psychological well-being of employees.

*H1: Safety specific transformational leadership is positively associated with the psychological well-being of employees*.

### Mediating Role of COVID-19 Perceived Risk

Occupational health researchers have identified safety specific leadership as a positive leadership style that decreases occupational hazards and makes employees feel safe at the workplace (Mullen and Kelloway, 2009). Multiple studies have shown that safety specific transformational leaders pay extra attention to employee safety, due to which employees start considering their workplace less hazardous (Conchie and Donald, 2009; Johnson, 2019; Smith et al., 2020). Safety specific transformational leader minimizes the risk of incidents and other unwanted events at the workplace (Willis et al., 2017). Safety transformational leadership is mainly studied in organizations exposed to occupational hazards, and employees working in these organizations revealed that they feel safe under a safety-specific transformational leader (Smith et al., 2016). Safety specific transformational leaders give priority to the safety of employees over the organizational goals, which may decrease COVID-19 perceived risk among healthcare workers.

The research on COVID-19 perceived risk suggests that preventive measures taken by the hospitals against COVID-19 help in minimizing the perceived risk associated with it (Ahmed et al., 2020; Bashirian et al., 2020; Wee et al., 2020; Zhao et al., 2020b). Since safety specific transformational leaders take extraordinary measures to ensure that healthcare workers don't contain the virus from patients, these measures likely decrease COVID-19 perceived risk among employees. Perceived risk of catching the infection is detrimental to the well-being of healthcare workers as they face this constant fear that their life is in danger and that they may also develop symptoms of COVID-19 (Gorini et al., 2020; Lam et al., 2020; Yan et al., 2020; Yildirim et al., 2020; Yildirim and Güler, 2021). On the other hand, a lower level of COVID-19 perceived risk can improve the mental health of employees (Cinar et al., 2020). The current studies have also shown that occupational hazards affect employee well-being, whereas workplace safety yields positive mental health outcomes for employees (Harrison, 2012; Amponsah-Tawiah et al., 2014; Hofmann et al., 2017; Alhassan and Poku, 2018; Chari et al., 2018).

The high reliability organizational theory (Roberts, 1990; La Porte, 1996) also supports the mediating role of perceived risk between safety specific transformational leadership and the psychological well-being of employees. This theory defines high reliability organizations as those organizations where extra focus is given to minimize occupational risks (Enya et al., 2018). Some researchers also state that high reliability organizations face minimum errors and risks due to their continuous efforts to promote safety. Employees working in these organizations show positive behaviors (Thomassen et al., 2011; Ford, 2018). Since safety specific transformational leaders transform employees by encouraging safe behavior at the workplace (Veazie et al., 2019), the COVID-19 perceived risk reduces as employees automatically start perceiving that their leader is making extra efforts to ensure their safety which enhances their psychological well-being. Hence, we propose the following hypothesis:

*H2: COVID-19 perceived risk mediates the relationship between safety specific transformational leadership and psychological well-being*.

### Moderating Role of Employee Safety Consciousness

The research on employee safety has identified the importance of developing a positive attitude toward safety (Remawi et al., 2011; Momani et al., 2017). Occupational health researchers suggest that leadership alone cannot prevent occupational health hazards; employees also need to play their role in making the workplace safer (Mullen et al., 2017; Shen et al., 2017; Koers, 2021). Due to the negative consequences of occupational hazards, organizations are looking for employees with a higher level of safety consciousness as it helps avoid hazards (Lee, 2017; Prussia et al., 2019; Meng and Chan, 2020).

According to a recent study, ethical leaders promote safety consciousness among employees, due to which organizational safety performance increases (Khan et al., 2018). Safety consciousness refers to an awareness of safety issues present at the workplace, present at the cognitive and behavioral level both (Barling et al., 2002). At a cognitive level, employees mentally feel mindful and attentive to safety issues present at the workplace, whereas safety consciousness at a behavioral level promotes safety behaviors at the workplace (de Koster et al., 2011). To summarize, employees with safety consciousness do not develop mental awareness regarding safety issues, but they also engage in safety procedures, which helps minimize the chances of injury or illness (de Koster et al., 2011; Lee, 2017; Prussia et al., 2019).

The handful of studies conducted on safety consciousness have failed to investigate its role in the event of a pandemic like COVID-19. The current study proposes that safety consciousness is an important individual factor, which, when joined with safety specific transformational leadership, helps mitigate COVID-19 perceived risk among healthcare workers. Other studies have also shown that the interactive effect of positive leadership style and safety consciousness yield positive outcomes (Mullen et al., 2017; Shen et al., 2017; Koers, 2021). Safety specific transformational leaders take solid actions to enhance workplace safety, whereas safety conscious employees display safety behaviors. Together, they minimized perceived COVID-19 risk among healthcare workers. When followers of safety specific transformational leaders have safety consciousness and are taking measures to ensure safety against COVID-19, then COVID-19 perceived risk automatically reduces. When the individual himself ensures safety and his/her leader also promotes safety protocols, the individual is less likely to risk catching the COVID-19 infection.

The moderating role of safety consciousness between safety specific transformational leadership and COVID-19 perceived risk gets its support from high reliability organizational theory, which focuses on risk prevention at the workplace (Roberts, 1990; La Porte, 1996). According to this theory, high reliability organizations are those organizations in which leaders take solid actions to minimize occupational risks and hazards by developing an action plan and following strict guidelines to enhance safety (Sujan, 2017). In addition, this theory also states that those high reliability organizations can minimize occupational hazards that create safety consciousness among the organizational members. These organizations enjoy positive employee outcomes (Ford, 2018). Since safety transformational leadership focuses on minimizing occupational risks and safety consciousness and promoting those behaviors that help reduce occupational risk, their interaction may help minimize COVID-19 risk up to a great extent. Hence, we propose that safety consciousness moderates the relationship between safety specific transformational leadership and COVID-19 perceived risk.

*H3: Safety consciousness moderates the relationship between safety specific transformational leadership and COVID-19 perceived risk such that the negative relationship will be stronger in case of high safety consciousness and weaker in case of low safety consciousness*.

## Methods

### Participants and Procedure

The current study is quantitative and time-lagged. Data were collected in three time lags with a gap of 3 weeks each through the questionnaire. Data for demographic variables, safety specific transformational leadership, and safety consciousness were collected at time 1. After a gap of 3 weeks, data for COVID-19 perceived risk were collected at time 2. Finally, data for psychological well-being were collected at time three after a gap of another 3 weeks (See Figure 1). A unique I.D. was assigned to each respondent, which was used to match the respondents' responses across all three-time intervals. Researchers believe that time-lagged research design has an advantage over cross-sectional research design as, unlike cross-sectional research, it minimizes common method bias (Podsakoff et al., 2012). Other studies have also used a time-lagged research design to minimize common method bias (Majeed and Fatima, 2020; Majeed et al., 2020).

Data were collected from those healthcare workers who were treating COVID-19 patients in different government and private hospitals of Pakistan. We collected data from only those healthcare workers who met the inclusion criterion, which required working in the COVID-19 ward in the hospitals and a minimum of 6 months of working experience as a full-time employee. The participation was done voluntarily, and we ensured participants that their data would be kept confidential. We contacted healthcare workers by using our contacts and collected the email addresses of those healthcare workers who showed their willingness to patriciate in the survey. The questionnaires were sent to their email address.

Data were collected between May 2020 and July 2020. The number of COVID-19 cases increased during the data collection period in Pakistan and worldwide. According to the Government of Pakistan's official website, 6,631,110 confirmed cases of COVID-19 were reported between February 2020 and June 2020, whereas 10,145 people lost their lives (Dil et al., 2020; Government of Pakistan, 2020; Yousaf et al., 2020). According to statistics shared by National Emergency Operation Center, more than 300 healthcare workers got affected by COVID-19, out of which 100 healthcare workers lost their lives to it (Junaidi, 2020). According to the Johns Hopkins Institute, more than three million people got COVID-19 between December 2019 and July 2020, whereas the figure reached 127,863,066 at the end of March 2021 (Johns Hopkins Institute, 2021).

The rule of thumb method is also called as N:q method, where *N* refers to cases or observations and *q* refers to the number of free parameters. The major rationale for choosing this method is that many researchers have recommended using this method to find the sample size for studies involving structural equation modeling (Bentler, 1995; Kline, 2005; Schreiber et al., 2006). The rule of thumb of 10 is the preferred rule of thumb compared to the rule of thumb of 5 (Boomsma and Hoogland, 2001; De Carvalho and Chima, 2014). Hence, we used the rule of thumb of 10. According to this rule, 10 responses are collected against each item. There was a total of 39 items in the survey, so a sample size of 390 was selected (39^*^10 = 390). Researchers widely use the rule of thumb to find the sample size (Hair et al., 2014, p. 100). Due to their demanding schedule, most of the healthcare workers refused to participate in the survey. We contacted only 327 healthcare workers at the time, one out of which 303 responses were received. We sent the survey to these 303 respondents at time two, but we received only 264 responses. At time 3, we sent the survey to 264 respondents who participated in time one and two. Out of these 264, 232 fully complete responses were received, which were used for data analysis. According to researchers, a sample size around *N* = 200 is sufficient for testing models involving structural equation modeling (Boomsma and Hoogland, 2001; Kline, 2005). Hence, our sample size is adequate.

We conducted a power analysis to make sure our final sample was appropriate. We used G^*^Power (version 3.1.9.4) for this purpose. A *Post hoc* analysis was done by setting predictors to three while keeping other parameters to default settings (i.e., α level = 0.05, the medium effect size of 0.15). The 232 sample size generate a high power of 0.99, which confirmed that the collected sample is adequate for testing the proposed model (Cohen, 1992; Faul et al., 2009; Memon et al., 2020).

Out of 232 respondents, 137 were female, whereas the remaining 95 respondents were male. Sixty-seven percent of respondents were between 25 and 35 years of age. One hundred twenty-four respondents had a nursing diploma or Bachelor's degree, 47 had a Master's degree, whereas 61 respondents contained MBBS degree. One hundred forty-seven respondents served as nurses, 61 respondents served as a doctor, and 24 respondents worked as paramedics staff. Fifty-seven percent of respondents had up to 5 years of working experience as a full-time employee.

### Measures

We adopted well-established scales for all the study variables. We distributed the questionnaire in English as it is the official language of Pakistan. Other researchers have also used the English language for collecting data from healthcare workers of Pakistan and did not face any language-related issue (Majeed and Fatima, 2020).

### Safety Specific Transformational Leadership

Data for safety specific transformational leadership were collected using a 10-item scale developed by Barling et al. (2002). All the items were measured using a five-point Likert scale ranging from 5 = Strongly Disagree and 1 = Strongly Agree. The sample item states, “My manager shows determination to maintain a safe work environment.” The Cronbach alpha for this variable is 0.91 in the current study.

### Psychological Well-Being

Data for psychological well-being were collected using an 18-item version of the scale developed by Ryff (1989). All the items were measured using a five-point Likert scale ranging from 5 = Strongly Disagree to 1 = Strongly Agree. The sample item states, “I like most parts of my personality.” The Cronbach alpha value for psychological well-being is 0.93.

### COVID-19 Perceived Risk

We used a 4 item scale developed as part of an extended parallel processing model (EPPM) by Witte (1996) to measure COVID-19 perceived risk. All the items were measured using a five-point Likert scale ranging from 5 = Strongly Disagree to 1 = Strongly Agree. A sample item is “I believed that I am at risk for getting COVID-19.” In the current study, the Cronbach alpha value for COVID-19 perceived risk is 0.80.

### Safety Consciousness

Safety consciousness was measured by using a scale developed by Barling et al. (2002). The scale contains seven items. All the items were measured using a five-point Likert scale ranging from 5 = Strongly Disagree to 1 = Strongly Agree. A sample item is “know what protective equipment and/or clothing is required for my job.” The value of Cronbach alpha for safety consciousness is 0.85 in the current study.

### Confirmatory Factor Analysis

Confirmatory factor analysis was performed to check the convergent and discriminant validity of the proposed model. The items loadings for all four factors were found higher than 0.60. The correlations between all the latent factors were also found in the acceptable range. Furthermore, four factors model also yielded better fit indices i.e., χ^2^ = 878, χ^2^/df = 1.26, IFI = 0.96, TLI =0.95, CFI = 0.96, RMR = 0.06, and RMSEA = 0.03 than one-factor model by loading all items on single factors i.e., χ^2^ = 2,325, χ^2^/df = 3.31, IFI = 0.61, TLI =0.58, CFI = 0.60, RMR = 0.12, and RMSEA = 0.10. The fit indices for the four-factor are in line with recommended model fitness criteria (Hu and Bentler, 1999; Hair et al., 2014). These results prove both convergent and discriminant validity of the proposed four factors model.

### Analysis of Variance

The current study collected data for age, gender, education, designation, and job experience in addition to study variables. We conducted an analysis of variance (ANOVA) test to identify those demographic variables which have a significant relationship with the study variables. Gender showed non-significant relationship with safety consciousness (*F* = 0.83, *P* = 0.36), COVID-19 perceived risk (*F* = 0.1.07, *P* = 0.30), and psychological well-being (*F* = 1.11, *P* = 0.29). Age also showed non-significant association with safety consciousness (*F* = 0.46, *P* = 0.63), COVID-19 perceived risk (*F* = 1.34, *P* = 0.26), and psychological well-being (*F* = 0.20, *P* = 0.81). The relationship between education and all study variables namely safety consciousness (*F* = 2.47, *P* = 0.08), COVID-19 perceived risk (*F* = 2.65, *P* = 0.07), and psychological well-being (*F* = 1.63, *P* = 0.19) was also non-significant. Experience also showed non-significant relationship with safety consciousness (*F* = 2.47, *P* = 0.08), COVID-19 perceived risk (*F* = 2.65, *P* = 0.007), and psychological well-being (*F* = 1.63, *P* = 0.19). Lastly, designation was also shown to have non-significant relationship with safety consciousness (*F* = 0.27, *P* = 0.59), COVID-19 perceived risk (*F* = 0.37, *P* = 0.54), and psychological well-being (*F* = 0.82, *P* = 0.36). These results show that none of the demographic variables shares a significant relationship with study variables. Hence, these variables were not controlled while conducting further analysis.

## Results

### Correlation

Table 1 shows the results of mean, standard deviation, correlation and reliability analysis. The Cronbach alpha value for all the variables is greater than the cutoff value of 0.70, which confirms the reliability of all the measures. The results of correlation showed that safety specific transformational leadership is significantly and negatively related with COVID-19 perceived risk (*r* = −0.30, *p* < 0.01) and significantly positively related to psychological well-being (*r* = 0.45, *p* < 0.01), and safety consciousness (*r* = 0.39, *p* < 0.01). COVID-19 perceived risk showed a significant and negative correlation with psychological well-being (*r* = −0.44, *p* < 0.01) and safety consciousness (*r* = −0.56, *p* < 0.01). Psychological well-being showed a positive and significant relationship with safety consciousness (*r* = 0.33, *p* < 0.01).

**Table 1:** Mean, standard deviation, reliability, and correlation.

**S. No**	**Variable**	**M**	**S.D**	**1**	**2**	**3**	**4**
1	SSTL	3.66	0.69	**(0.91)**			
2	COVID-19 perceived risk	3.29	0.89	−0.30^**^	**(0.80)**		
3	Psychological well-being	3.41	0.72	0.45^**^	−0.44^**^	**(0.93)**	
4	Safety consciousness	3.52	0.73	0.39^**^	−0.56^**^	0.33^**^	**(0.85)**

*N = 232*,

* *p < 0.05*,

** *p < 0.01*;

*Cronbach alpha are provided bold in parentheses*.

*SSTL, Safety Specific Transformational Leadership*.

### Direct and Indirect Effect

Table 2 shows the results of direct effect and indirect effect. We used model 4 of Process Macro by Hayes for testing the mediation hypothesis. The results of direct effect showed a significant relationship between safety specific transformational leadership and psychological well-being (β = 0.36, *p* < 0.01), which led to the acceptance of hypothesis 1. The relationship between safety specific transformational leadership and COVID-19 perceived risk was also negatively significant (β = −0.39, *p* < 0.01), whereas COVID-19 perceived risk also showed a negative and significant relationship with psychological well-being (β = −0.28, *p* < 0.01). The Bootstrap 5000 results of the indirect effect of safety specific transformational leadership on psychological well-being through COVID-19 perceived risk were also significant at 95% confidence interval (*Indirect effect* = 0.11, *LL* = 0.07, *U.L*. = 0.17). The upper and lower limits 95% confidence intervals contain no zero. Hence, hypothesis 2 is also supported.

**Table 2:** Bootstrapping results for direct and indirect effects.

**Direct effect**	**Effect**	**S.E**	***t***
SSTL → psychological well-being	0.36^**^	0.06	5.89
SSTL → COVID-19 perceived risk	−0.39^**^	0.08	−4.80
COVID-19 perceived risk → psychological well-being	−0.28^**^	0.04	−5.99

*N = 232*,

* *p < 0.05*,

** *p < 0.01*.

*SSTL, Safety Specific Transformational Leadership; LL, Lower limit; UL, Upper limit; CI, Confidence interval; S.E, Standard error*.

### Moderation Analysis

**Figure 2:**
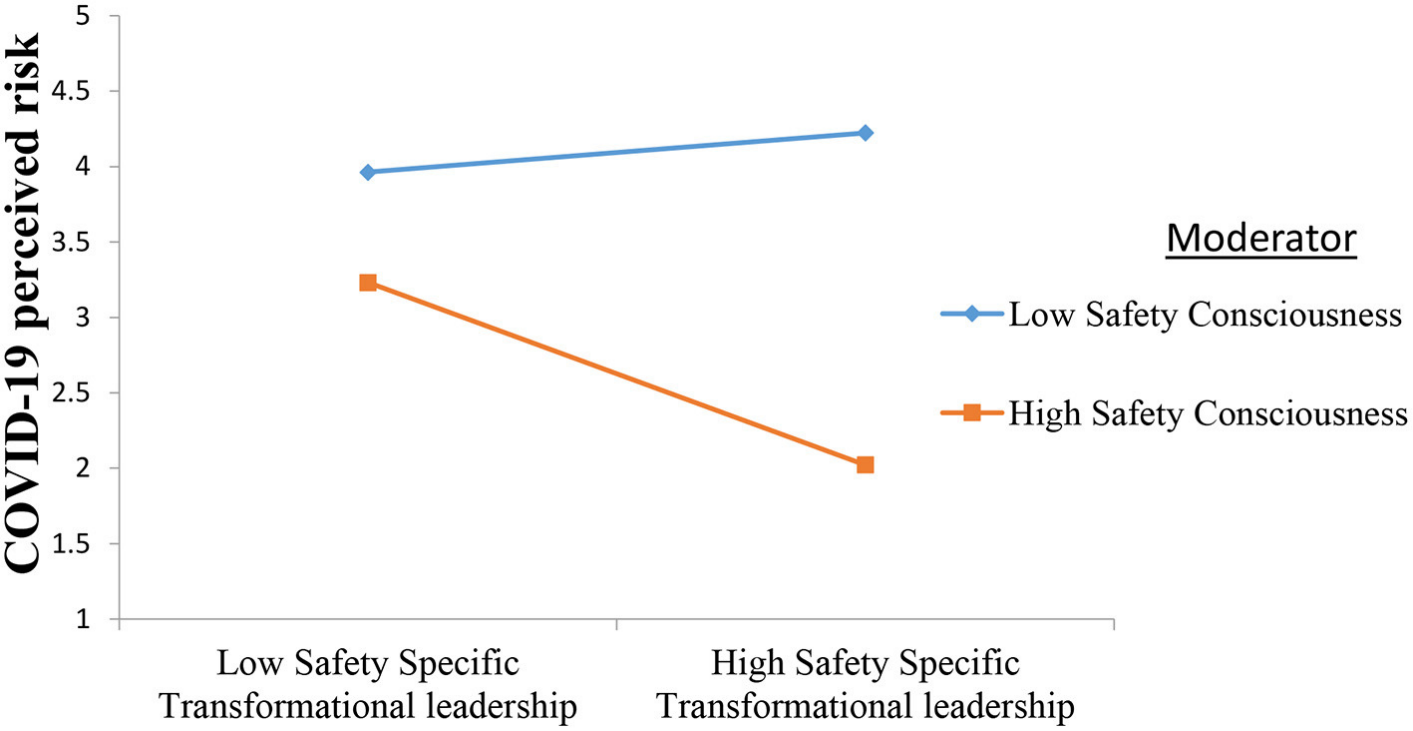
Moderating role of safety consciousness. Shows that safety consciousness strengthens the negative relationship between safety specific transformational leadership and COVID-19 perceived risk.

We used model 1 of Process Macro by Hayes to test the moderating role of self-consciousness between safety specific transformational leadership and COVID-19 perceived risk. The rationale for using Process Macro for moderation is that it also gives results for the slope test by showing the variance in the relationship between independent and dependent variables at high, medium and low values of moderator. Keeping in view the recommendations of Aiken et al. (1991), safety specific transformational leadership and safety consciousness were centered around mean. The interactive effect of safety specific transformational leadership and safety consciousness on COVID-19 perceived risk was negative and significant (β = −0.37, *p* < 0.01). The R square change was also significant for the interactive effect (Δ*R*^2^ = 0.06, *p* < 0.01). The slope test results further confirmed that the negative relationship between safety specific transformational leadership and COVID-19 perceived risk strengthens at high values of safety consciousness (β = −0.50, *LL* = −0.12, *U.L*. = −0.72). Hence, hypothesis 3 is also accepted. Figure 2 shows the graph for moderation. The graph also confirms that safety consciousness moderates the negative relationship between safety specific transformational leadership and COVID-19 perceived risk such that this relationship is stronger at high safety consciousness than low. Hence, hypothesis 3 of the study was also supported. The moderation graph is also presented. Table 3 shows the results of moderation analysis.

**Table 3:** Moderation analysis.

	**Safety consciousness**		
	**β**	**S.E**	***ΔR^2^***
Constant	3.36		
SSTL → COVID-19 perceived risk	−0.24^**^	0.07	
Safety consciousness → COVID-19 perceived risk	−0.73^**^	0.07	
SSTL × safety consciousness → COVID-19 perceived risk	−0.37^**^	0.07	0.06^**^

*N = 232*,

* *p < 0.05*,

** *p < 0.01*.

*SSTL, Safety Specific Transformational Leadership; LL, Lower Limit; UL, Upper limit; CI, Confidence interval; SD, Standard deviation; M, Mean; S.E, Standard error*.

**Figure 3:**
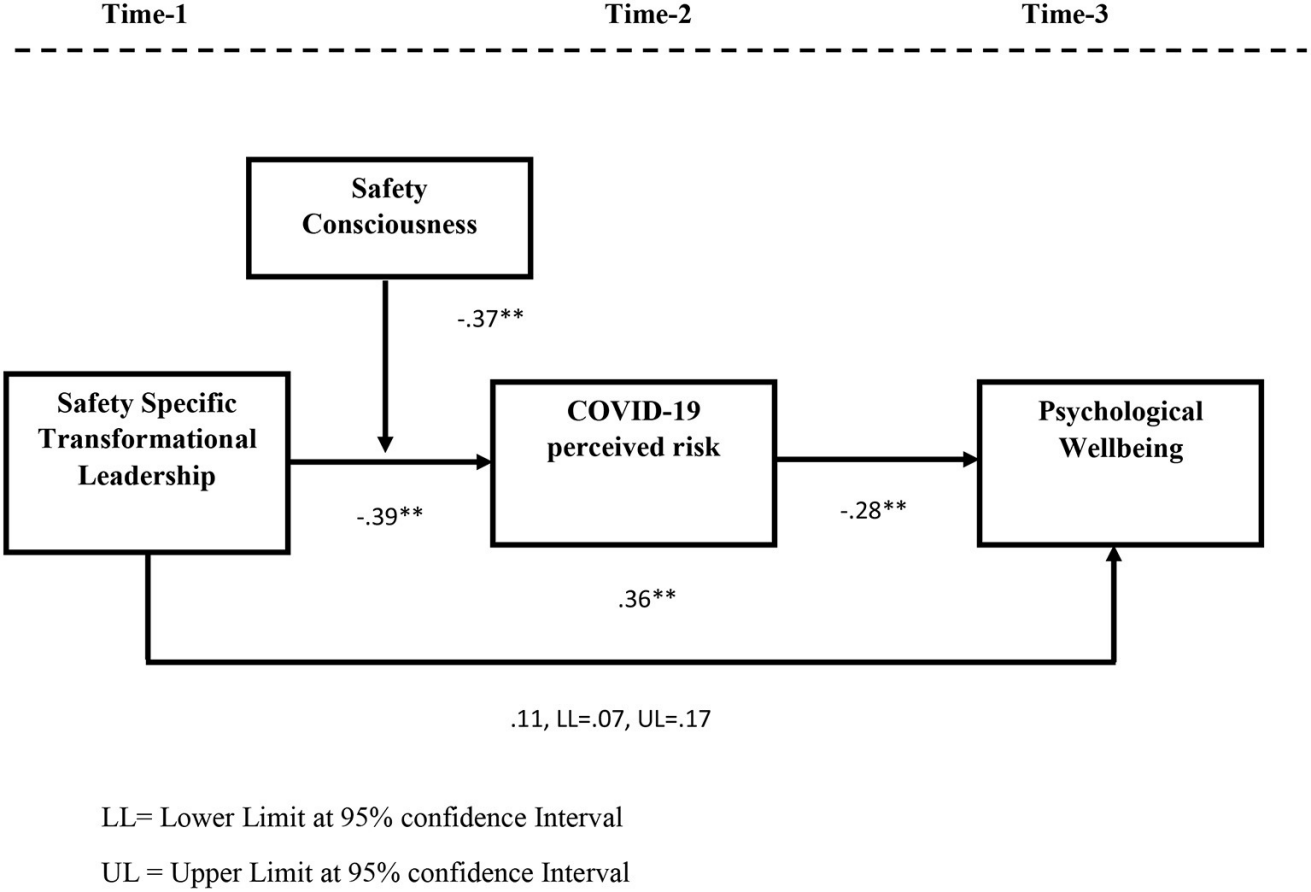
Diagrammatic representation of bootstrapping results for mediation and moderation. LL, Lower Limit at 95% confidence Interval; UL, Upper Limit at 95% confidence Interval. Shows bootstrapping results for direct and indirect effects where safety specific transformational leadership enhances psychological well-being of employees by reducing COVID-19 perceived risk.

Figure 3 shows the diagrammatic representation of mediation and moderation results.

## Discussion

After more than a year, the number of COVID-19 cases is still rising (Verelst et al., 2020; Venkatesan, 2021). Millions of people worldwide, including many healthcare workers, have lost their lives to it, while many are still fighting against it (Erdem and Lucey, 2021; Lapolla et al., 2021). COVID-19 is a serious workplace hazard that has drained healthcare workers physically and mentally (Gohar et al., 2020; Nguyen et al., 2020). Although providing hundred percent protection to healthcare workers against COVID-19 is inevitable due to their frequent and direct exposure to COVID-19 patients, hospital management can make hospitals a safer place for healthcare workers (Wee et al., 2020; Zhao et al., 2020b). Keeping this in view, we proposed that hospitals need safety specific transformational leadership during this pandemic and safety-conscious employees as both these factors together help in reducing COVID-19 perceived risk. This decreased in risk leave a positive impact on healthcare workers by enhancing their psychological well-being.

The results supported our theoretical framework, leading to the conclusion that safety transformational leadership and safety consciousness among employees decrease COVID-19 perceived risk, leading to an increase in the psychological well-being of healthcare workers. These results are consistent with existing studies which have shown that safety specific leadership promotes perceived safety and other positive outcomes among employees (Conchie and Donald, 2009; Johnson, 2019; Smith et al., 2020) and a decrease in COVID-19 perceived risk causes improvement in the mental health of employees when hospital management take soli measures to ensure the safety of healthcare workers (Ahmed et al., 2020; Bashirian et al., 2020; Dirani et al., 2020; Zhao et al., 2020b; Haque, 2021).

### Theoretical and Practical Implications

The current study has several theoretical strengths. First, it advances research on healthcare workers treating infectious diseases in general and COVID-19 in particular. It is amongst the pioneer studies to investigate the outcomes of safety specific transformational leadership in the context of the pandemic by suggesting it to be the most appropriate leadership style for managing healthcare workers who are providing treatment against infectious diseases. The existing studies have mostly linked it to occupational safety and workplace hazards. It has also shed light on the role of leadership in decreasing COVID-19 perceived risk and increasing psychological well-being among healthcare workers. It also has extended research on safety consciousness in the context of a pandemic.

The results of the study offer implications for hospital managers. Hospitals must encourage the hospital managers to adopt safety-specific transformational leadership to minimize healthcare workers' safety-related concerns and enhance their psychological well-being. This may require the managers to shift their focus from meeting organizational goals to ensuring the safety of healthcare workers. Managers must also encourage healthcare workers to provide suggestions for enhancing safety which may involve abandoning any outdated safety procedure or following more effective safety guidelines. Managers must also give individual consideration to the safety of healthcare workers by making sure every healthcare worker is safe.

Most importantly, they should act as a role model by following safety protocols and encouraging others to follow the standard safety procedures. Hospital management must also provide safety training to the healthcare workers to enhance their safety consciousness. Healthcare workers should be closely monitored to make sure they are strictly following the safety protocols. Hospitals must share updated information on safety procedures with employees from time to time to enhance their safety consciousness level. The risks of violating the safety protocols should also be shared with the staff to enhance their safety consciousness.

Hospitals must introduce programs designed to enhance the psychological well-being of healthcare workers during this critical time. For instance, Resilient in Stressful Events (RISE) and Second Victim Experience Support Tool (SVEST) help reduce stress caused due to work and non-work related issues (Scott, 2009; Migdole et al., 2011; Burlison et al., 2017; Connors and Wu, 2020). On a more general level, it is crucial to develop a supportive organizational culture (Higgins, 2015) by adopting a peer support program, as suggested by De Clercq et al. (2020), in which senior healthcare workers are asked to encourage fellow employees with self-care tips and psychological first-aid (Migdole et al., 2011). These programs have the purpose of providing peer support to employees who are facing any mental health issues. They comprise different activities such as giving emotional support, discussing workplace practices, listening to employees' issues, and creating a supportive environment (Scott, 2009; Migdole et al., 2011).

### Limitations and Future Research Directions

The results of the current study should be viewed in light of its limitations. The current study only investigates the psychological outcome of safety specific transformational leadership. Future studies have investigated its performance-related or behavioral outcomes. Another limitation of this study is that it has taken employee perception as an underlying mechanism between safety specific transformational leadership and employee psychological well-being. Future researchers may test the mediating role of employee attitudes and emotions between safety specific transformational leadership and employee psychological well-being. This study has not taken any personality disposition as the boundary condition. Future studies can extend this study by investigating the moderating role of different personality traits and situational factors. The current study followed a time-lagged research design which offers benefits against cross-sectional research design but fails to catch variance among study variables over time. Future studies may replicate the findings of this study by collecting longitudinal data to test the change in variance among proposed variables across time. Lastly, the results of this study are only limited to healthcare workers. Future researchers may conduct studies on employees working in other sectors in which employees are required to remain physically present at the workplace during COVID-19, such as banks.

## Conclusion

Healthcare workers have become frontline soldiers against COVID-19. However, they are under immense stress due to frequent exposure to infected patients. The current study has identified safety specific transformational leadership as a suitable leadership style that combines with employee safety consciousness and dampens the COVID-19 perceived risk among healthcare workers. This reduction in perceived risk improves psychological well-being among healthcare workers.

## Data Availability Statement

The raw data supporting the conclusions of this article will be made available by the authors, without undue reservation.

## Ethics Statement

The studies involving human participants were reviewed and approved by The National University of Modern Languages (NUML) constitutes the departmental Ethics Approval Committee. The Faculty of Management Sciences, NUML Research Ethics Board, reviewed The Combined Effect of Safety Specific Transformational Leadership and Safety Consciousness on Psychological Well-being of Healthcare Workers research proposal and considers the procedures, as described by the applicant, to conform to the University's ethical standards and university guidelines. Moreover, the participation in the survey was voluntary, and study participants were first explained about the details of the project. It was assured to them that their responses will be kept in strict anonymity and will be reported as aggregate results. The patients/participants provided their written informed consent to participate in this study.

## Author Contributions

All authors listed have made a substantial, direct and intellectual contribution to the work, and approved it for publication.

## Conflict of Interest

The authors declare that the research was conducted in the absence of any commercial or financial relationships that could be construed as a potential conflict of interest.
